# A case of misalignment: the perspectives of local and national decision-makers on the implementation of psychological treatment by telephone in the Improving Access to Psychological Therapy is the name of the service and should be capitalised

**DOI:** 10.1186/s12913-019-4824-4

**Published:** 2019-12-26

**Authors:** Kelly Rushton, Claire Fraser, Judith Gellatly, Helen Brooks, Peter Bower, Christopher J. Armitage, Cintia Faija, Charlotte Welsh, Penny Bee

**Affiliations:** 1 School of Health Sciences, Division of Nursing, Midwifery and Social Work, University of Manchester, Manchester Academic Health Science Centre, Manchester, UK; 2 Department of Health Services Research, Institute of Population Health Sciences, University of Liverpool, Liverpool, UK; 3 NIHR School for Primary Care Research, Centre for Primary Care and Centre for Health Informatics, Manchester Academic Health Science Centre, University of Manchester, Manchester, UK; 4 School of Health Sciences, Division of Psychology and Mental Health, University of Manchester, Manchester University NHS Foundation Trust, Manchester Academic Health Science Centre, Manchester, UK; 5 School of Health Sciences, Division of Psychology and Mental Health, University of Manchester, Manchester Academic Health Science Centre, Manchester, UK

**Keywords:** Telephone therapy, Psychological therapy, Guided self-help, Anxiety, Depression, Psychological wellbeing practitioner, IAPT, Mental health, Normalisation process theory, Implementation

## Abstract

**Background:**

Psychological treatment delivered by telephone is recommended by the National Institute for Health and Care Excellence (NICE) for mild to moderate depression and anxiety, and forms a key part of the Improving Access to Psychological Therapy (IAPT) programme in the UK. Despite evidence of clinical effectiveness, patient engagement is often not maintained and psychological wellbeing practitioners (PWPs) report lacking confidence and training to deliver treatment by telephone. This study aimed to explore the perspectives of professional decision makers (both local and national) on the barriers and facilitators to the implementation of telephone treatment in IAPT.

**Methods:**

Sixteen semi-structured qualitative telephone interviews and one focus group were carried out with decision makers (*n* = 21) who were involved locally and nationally in policy, practice and research. The interviews and focus group were coded thematically, and then mapped onto the four core constructs of Normalisation Process Theory (NPT).

**Results:**

The use of telephone for psychological treatment was universally recognised amongst participants as beneficial for improving patient choice and access to treatment. However, at service level, motives for the implementation of telephone treatments are often misaligned with national objectives. Pressure to meet performance targets has become a key driver for the use of telephone treatment, with promises of increased efficiency and cost savings. These service-focussed objectives challenge the integration of telephone treatments, and PWP acceptance of telephone treatments as non-inferior to face-to-face. Ambivalence among a workforce often lacking the confidence to deliver telephone treatments leads to reluctance among PWPs to ‘sell’ treatments to a patient population who are not generally expecting treatment in this form.

**Conclusions:**

Perceptions of a need to ‘sell’ telephone treatment in IAPT persist from top-level decision makers down to frontline practitioners, despite their conflicting motives for the use of telephone. The need for advocacy to highlight the clinical benefit of telephone treatment, along with adequate workforce support and guidance on best practice for implementation is critical to the ongoing success and sustainability of telephone treatment in primary care mental health programmes.

## Background

Psychological therapy delivered by telephone is recommended by the National Institute for Health and Care Excellence (NICE) as a treatment for mild to moderate depression and anxiety and forms a key part of the Improving Access to Psychological Therapy (IAPT) programme in the UK [1, 2]. The IAPT model, implemented in 2008 [3], is grounded in the principle of providing increased access to evidence-based interventions without compromising clinical outcomes and cost-effectiveness. Since its inception, the IAPT programme has inspired the introduction of other stepped-care models of psychological treatment, such as the NewAccess programme in Australia, which has demonstrated encouraging clinical and economic effectiveness [4, 5].

Research evidence for the clinical effectiveness of telephone interventions across differing illness severities has demonstrated superior effects to usual care depression [6], comparable clinical effectiveness compared to face-to-face treatment for depression in primary care [7] and as a clinically effective addition to antidepressant medication [8]. Similar evidence exists in relation to telephone treatments for anxiety in older adults [9], adults and young people with obsessive compulsive disorder [10, 11] and for co-morbid mental and physical health conditions [12, 13].

Analyses have demonstrated the cost-effectiveness and efficiency of telephone-delivered psychological interventions via lower service costs, reduced treatment time and reduced need for travel and treatment space [10, 14, 15]. Additional advantages for patients include flexibility, accessibility, anonymity and increased choice [16–18]..

However despite the evidence for clinical and cost-effectiveness, and emerging evidence in relation to the acceptability of telephone-delivered services [17–19], uptake of telephone delivered treatments by services remains variable. Although evidence exists from observational studies for effectiveness and cost-saving [15], much of the evidence supporting telephone interventions is derived from randomised controlled trials (RCTs), in which therapists have often received bespoke telephone-specific training, and who may not possess the same attitudes towards telephone working as those in clinical practice [7–9]. Where telephone treatment has been implemented in practice, organisational support for telephone working is often insufficient for psychological wellbeing practitioners (PWPs) [18, 20], and patient engagement has been poor compared to face-to-face treatment [21].

While previous studies have highlighted some of the potential barriers to the use of telephone treatment at a clinical level [17, 18, 22], these accounts do not afford insight into the wider influences of decision-makers. Recognising the importance of context and multiple levels of influence is an important element in professional behaviour change theory and health service innovation [23, 24].

This study aimed to address this gap by exploring the views of local and national decision makers about the barriers and facilitators to implementation of telephone treatments in IAPT. These decision makers were involved in regional and national policy, local clinical leadership and practice, and research in IAPT. Engaging with such individuals offers unique perspectives to gain insight about the wider contextual issues and political and policy agendas impacting on the implementation of telephone treatments from the ‘top down’ [25, 26].

Exploring the views of decision makers will provide the opportunity to examine how local implementation aligns with national policy, and how national objectives cascade down through the IAPT model to the clinical interface. We have used this approach successfully in other research exploring decision maker perspectives on innovative policy implementation and variability at local levels within mental health services [27]. The current study is part of a wider research programme aiming to enhance the delivery of psychological treatments by telephone in IAPT services [28].

### Using normalisation process theory to understand the current context

Telephone is a recommended mode of delivery for psychological treatments for mild to moderate depression and anxiety [1, 2], and as such it is important to develop a comprehensive understanding of the challenges that face services when integrating telephone treatments into the standard offer for patients accessing IAPT services. Normalisation Process Theory (NPT) is an evidence-based theoretical approach to understanding implementation processes and the factors needed for successful integration of interventions into existing practice [29], and has been used across multiple fields, including mental health [30, 31]. NPT consists of four main constructs which can be used to explore the factors, both individual and group, that might promote or inhibit the ‘normalisation’ of telephone-delivered psychological interventions (Table 1).

**Table 1:** Normalisation Process Theory constructs

Construct	Application to current study
Coherence	The sense-making, meaning or understanding regarding telephone delivered psychological interventions. An exploration of decision makers’ understanding of the purpose and potential value of telephone treatments.
Cognitive Participation	The level of engagement, commitment or buy in. The relational work needed to enhance the delivery of psychological interventions by phone, what decision makers can contribute to ensure commitment.
Collective Action	The action or operational work needed to be undertaken to support the implementation of telephone delivered psychological interventions. What resources are required to make it work in practice?
Reflexive Monitoring	The formal and informal appraisal which takes place; the assessment of the advantages and disadvantages and impact post-implementation and whether improvements can be made to support sustainability.

NPT has been used previously to explore the perspectives of cognitive behavioural treatment (CBT) therapists on the embedding of telephone work in higher intensity CBT treatment [22]. The current study is the first to apply NPT principles to the exploration of decision maker’s perspectives of the policy and organisational issues regarding the implementation of telephone as a mode of treatment in a psychological stepped care programme.

## Methods

### Study design and recruitment

The study was designed to seek the views of decision makers who could provide insights into policy and organisational issues concerning implementation of telephone treatments. We sought to identify those involved locally and nationally in policy, practice and/or research in relation to IAPT service delivery and workforce development. Potential participants were identified by reviewing IAPT policy documentation, consulting with the Programme Steering Committee (individuals with expertise in a variety of areas appointed to oversee the research programme), via existing research team contacts with National Health Service (NHS) and third sector services and via snowball sampling. As each participant was considered to be an expert in their own right with specific expertise in relation to national policy and local contexts, we achieved information power [32] by seeking the views of key individuals related to the IAPT programme.

Potential participants were sent an email with a participant information sheet explaining the study, and a consent form to be signed and returned in advance of interviews taking place or for collection on the day for focus group participants. The study was approved by North West Greater Manchester West Research Ethics Committee (Ref: 18/NW/0372).

### Participants

Thirty five people were invited to take part, and twenty-one (60%) went on to be interviewed (11 females, 52%)). Two individuals declined as a result of having no time, and there were 12 non-responders. Participants included national policy and/or academic leads (*n* = 6); local NHS service managers/clinical leads (*n* = 9); and managers/clinical leads of local 3rd (voluntary) sector commissioned services (n = 6). Professional experience ranged from 1 to 20+ years. Local informant role responsibilities included clinical practice, clinical leadership, service management and decision making around front-line practice, PWP working arrangements and service provision of telephone treatment. National informant responsibilities encompassed education and curriculum, national policy decisions, academic and research activity. All but one participant had experience of delivering psychological therapies in addition to their current policy or management roles (Table 2).

**Table 2:** Participant details for data contextualisation (CBT – Cognitive Behavioural Therapy, DBT – Dialectical Behavioural Therapy, EMDR - Eye Movement Desensitisation Reprogramming, HIT – High Intensity Therapy)

Participant	Position	Current Role	Clinical Training	Experience	Level of current service provision of telephone treatment
1	Local	Service manager	No	N/A	Yes
2	National		CBT	Telephone/face-to-face/group/bibliotherapy	N/A
3	Local	Service manager/clinical lead	CBT	Telephone/face-to-face/bibliotherapy/online	Yes
4	National		CBT	Telephone/Face-to-face	N/A
5	Local	Clinical lead	Clinical psychology	Face to-face/group	Yes
6	Local	Trust lead	CBT/EMDR	Face-to-face	Yes
7	Local	Clinical lead	CBT	Face-to-face/group	Yes
8	National		CBT	Face-to-face	N/A
9	Local	Clinical lead/psychologist	Clinical psychology	Face-to-face	Yes
10	National		CBT/ Interpersonal	Telephone/face-to-face/online	N/A
11	Local	Clinical lead	Clinical psychology	Telephone/face-to-face/group/online/bibliotherapy	Yes
12	National		CBT	Telephone/face-to-face	N/A
13	Local	Service manager	CBT/Psychodynamic		
14	National		HIT/CBT	Face-to-face	N/A
15	Local	Step 2 lead	PWP	Telephone/face-to-face/group/online	Yes
16	Local	Team manager	CBT	Telephone/face-to-face/group/online	Yes
17	Local	Deputy clinical lead	CBT	Telephone/Face-to-face	Yes
18	Local	Clinical lead	CBT/DBT/Psychodynamic	Face-to-face/group	Yes
19	Local	Step 2 lead	HIT	Telephone/face-to-face	Yes
20	Local	Step 3 lead	HIT	Face-to-face	Yes
21	Local	Step 2 lead	HIT	Face to face	Yes

### Procedure

Sixteen semi-structured interviews and one focus group (*n* = 5) were undertaken by an experienced female qualitative researcher (CF) with no involvement in IAPT service delivery or development. Interviews were digitally audio recorded and transcribed verbatim. All data collection took place between July and October 2018. Duration of interviews ranged from 21 to 54 min (focus group duration 52 min). The focus group participants were representatives of one service who requested this method of participation during their scheduled management team meeting slot, which took place at their service. All but one interview was completed by phone, and all participants were aware that CF was an experienced qualitative researcher.

The focus group and interviews were conducted concurrently, and followed a semi-structured schedule (Additional file 1) shaped around the four key constructs of NPT (coherence, cognitive participation, collective action, reflexive monitoring) [33] which explored participants’ perceptions and experiences of psychological interventions delivered by telephone, including reflections on local implementation, the national policy context and future sustainability of this treatment modality.

### Analysis

Data from interviews and the focus group were analysed in two stages. In the first stage of the analysis, transcripts were analysed thematically using the six-step process outlined by Braun and Clarke [34]. CF and KR initially familiarised themselves with the data by reading and re-reading transcripts, and initial codes were generated by CF who coded the data inductively using NVivo software [27]. Codes were then verified by KR, and the two researchers met to discuss emerging themes by identifying similarities, differences and relationships between codes. Themes were then reviewed to develop a preliminary thematic framework.

The second stage of the analysis involved the wider research team (CF, KR, JG, HB, PBee), who met to gain deeper insight of the data and refine themes to ensure accurate reflection of participant data. Any disagreement between researchers were addressed as a group and resolved through discussion to reach agreement. Themes and constituent codes were then mapped to the four constructs of the NPT framework [33]. To confirm accuracy, transcripts were then re-read a final time by CF. All themes were mapped to the framework and demographic information collected for participants was used to contextualise the data.

CF (MSc) is a Health Researcher, KR (PhD) is a Mental Health Researcher, JG (PhD) is a Programme Manager and Mental Health Researcher, HB (PhD) is a Lecturer and Health Service Researcher, PBee (PhD) is a Professor in Applied Health Research. All authors are currently involved in a wider programme of research aiming to enhance the quality of telephone delivered interventions in psychological services. Authors were conscious of their position in regard to the research topic and worked to minimise the risk of bias during data collection and analysis. No prior relationship existed between the research team and participants. Study participants had full understanding of the purposes of the research to establish their understanding and opinions of telephone treatments in IAPT.

## Results

The identified themes from the first stage thematic analysis were mapped onto the NPT framework and are presented in Table 3 and below using the four key constructs: Coherence, Cognitive Participation, Collective Action and Reflexive Monitoring. To provide context, the level of provision of telephone delivered treatments provided by the services of local informants is indexed for individual quotes. Level of provision is defined as ‘established’ (extensive use of telephone in service for assessment and treatment by all PWPs); ‘limited’ (infrequent use by a limited number of PWPs, often as replacement face-to-face sessions); ‘emergent’ (recent implementation of telephone use in individual teams, small numbers of PWPs using telephone, intention for greater integration of telephone treatment in service).

**Table 3:** Study findings presented using NPT core constructs

NPT construct	Study themes
Coherence	Barriers e.g. staff resistance Context e.g. service delivery model Facilitators e.g. accessibility and choice National policy context e.g. consistent protocol adherence Organisational factors e.g. tool to meet targets/save money Patient perspectives e.g. expectations ‘Selling’ telephone interventions e.g. to staff (incentives)
Cognitive Participation	Barriers e.g. engagement Facilitators e.g. organisational perspectives Patient perspectives e.g. patient resistance
Collective Action	Barriers e.g. I.T. systems Facilitators e.g. standardisation of approach PWP training e.g. in house training Resources to support telephone working e.g. working locations ‘Selling’ telephone interventions e.g. to patients
Reflexive Monitoring	Barriers e.g. facility to monitor mode of delivery Facilitators e.g. demonstrable efficiency Quality assessment e.g. patient experience questionnaire Sustainability e.g. use of technology

### Coherence: understanding and making sense of telephone-delivered psychological interventions

Typically, increasing engagement with telephone treatment for depression and anxiety in primary care relies on decision makers such as policy leads and service managers understanding its purpose and potential value, and effectively communicating that down to clinical services.

Participants in the current study displayed consensus in their views of the national policy context supporting the use of telephone treatment, and felt there was a good evidence base to support this position. However, policy documentation was considered to lack clear guidance about implementation at a local level causing regional variations in telephone use.

Although local autonomy and needs assessment were valued, there was felt to be a role for more national policy guidance around implementation, although many were keen to stress this should not be too prescriptive. Local decision makers discussed a lack of guidance on ‘best practice’ in telephone work to support local implementation:

*‘I don’t think they were that prescriptive about the telephone use. Indeed with the curriculum it basically says telephone use should be made available, it’s one of the choices and you should offer it but then it doesn’t go beyond that mention. And potentially the implementation in services it says much the same and leaves it to the service to decide themselves about how best to do it.’ (Participant 11, local informant, established use).*

The need to offer increased choice, flexibility and personalised care to patients in line with IAPT core aims were the principal drivers highlighted by national decision makers who spoke about how telephone treatments should be part of a *‘suite of different ways’* to deliver NICE approved evidence based treatments. The importance of personalised care was also considered important, with telephone treatment offered as an option to provide a way to deliver choice to patients who want such flexibility.

Patient centred drivers of telephone were also recognised and discussed by local decision makers, however service centred motives, such as cost-saving, were a predominant focus of some interviews with local service and clinical leads. The decision to offer telephone treatment was perceived to facilitate services to overcome financial and associated practical challenges, such as hiring costly clinic space in GP surgeries. It was also seen to provide increased ‘clinical efficiency’ by delivering cost savings to services through the use of telephone assessments, by minimising waiting lists, and by reducing the number of missed appointments:

*‘It's largely doing the telephone assessment, it has been a local decision that we've made with the CCG[clinical commissioning group] that…well a service would be a lot more costly if we were to do all face to face assessments. I think there's…I suppose there's a potential saving in doing telephone therapy, but it just boils down to practically whether we can, you know, realise some cash from that saving.’ (Participant 5, local informant, limited use)*

Telephone delivery was also considered at the national level as having a variety of advantages for PWPs, and subsequently services. The increased opportunity for flexible working and greater variety for PWPs was identified as important for tackling burnout in the IAPT workforce, along with providing PWPs with a tool to more easily manage and contain sessions. Decision makers acknowledged the importance of providing varied working options such as working from home to support PWPs, noting the potentially *‘draining’* experience of sitting in the same room everyday on the phone. The potential for telephone delivery to facilitate PWPs to contain sessions was also discussed in terms of adhering to agendas while at the same time preventing the perception of being over-managed, contributing to efficiency:

*‘So way back when I was a clinical lead…and as a therapist myself, I’d found that telephone and Skype sessions had really beneficial effects, that people seemed to be contained more within the sessions. I felt that agendas could be very strictly adhered to without feeling like you’re being over-managed. I think they can reduce the time spent in a session. Not because the therapist is trying to cut down the session, but because something happens by telephone and by Skype where some of the sessions become incredibly well-structured.’ (Participant 8, national informant)*

Whilst it could be argued that both patient and service-centred drivers are important, some higher level decision makers felt the two could be in conflict, where service needs, such as meeting performance targets, are balanced against clinical need and policy mandates around patient choice:

*“So from a clinical point of view, I can see that there’s a real choice agenda in widening access. From a, I guess, a service perspective, you’ve also got the issue of how do you meet the increasing targets and to increase access to meet a greater, higher level of prevalence. So the KPI’s [key performance indicators], also, I think, drive the telephone agenda but not necessarily for the same clinical reasons as choice and I think there’s a real tension there… when you’ve got service and performance needs being balanced against clinical need and I think telephone working can start to be used to meet service needs, rather than clinical needs, if that makes sense. That’s my…I think there’s a real tension there.” (Participant 4, national informant)*

The understanding of the potential for telephone to offer greater benefit to services rather than patients filtered down to local decision makers, who acknowledged it as a potential barrier to acceptance of telephone use by PWPs. Local decision makers talked about the importance of managing staff expectations, suggesting that persuading the clinical team of the benefits of telephone working was potentially a bigger challenge than convincing patients. This was particularly important in relation to the views that PWPs often feel they are short-changing patients by providing them with treatment which is only offered for financial reasons:

‘*I think the staff sometimes feel that they're short changing the patient by doing telephone and again it's trying to shift that attitude that it's not a financially driven thing, it's not about any other aspect other than is it appropriate to do so with this person.’ (Participant 3, local informant, emergent use)*

In terms of practicality for PWPs and services, there was additional divergence in views. While there was the view by some that a quiet space is essential for PWPs to work effectively on the telephone *‘You can’t do it in a busy office’ (Participant 16, local informant, limited use*’), a number of national decision makers held the opinion that PWPs would be better optimised to work by telephone in an open-plan call centre setting; an idea which aligns with the original IAPT model:

‘*I can't see how you can effectively do telephone work unless you’ve got a room like that [call centre]. That’s the way it’s designed to be done. Of course then you have a network of people, so you may have eight people in the room, six people in the room whatever, but you have a group of people who are all doing telephone work, they can support each other and it gives a model of how the whole thing is done.’ (Participant 10, national informant)*

Local informants from five different NHS trusts spoke of ‘call centre set-ups’ within their services, and the service benefits to such a system i.e. cost savings on estates, along with some of positives for PWPs (i.e. peer support). However, all those with call centre arrangements also acknowledged negative aspects for both PWPs and patients, most pertinently noise and distraction, which was suggested can affect the way patients perceive how they are valued. The ‘ideal’ provision of quiet spaces was expressed more prominently by local informants in services where less telephone work was undertaken, and by some national informants which contrasted sharply against other national informant views of the call centre environment being ‘the ideal’. The common denominator underscoring diverged views on such practical issues was actual experience; national informants had no personal experience of working in such environment’s leading opinions to be based on personal or policy views. Such disparity in opinions of key decision makers on matters central to the implementation of telephone may provide insight into the reasons for such variation in working practices across services, where local opinions may have more influence in the absence of national guidance.

Whilst some barriers to successful implementation were noted, telephone treatments were on the whole understood to facilitate greater accessibility and choice for a wide variety of patients (such as those with work and childcare commitments, people restricted by location, transport and mobility issues, and those with co-morbid/long term conditions). One participant also highlighted the potential for telephone interventions in tackling shame and stigma:

*“One thing I’d say is that telephone for me works well for people with high shame-based presentations…[they] might not want to come into a place and be around lots of people, and so it might be really useful to do that on the phone…they might not want to be identified. So I think there’s an advantage to engaging with hard to reach populations on that.” (Participant 8, national informant)*

Although national decision makers felt that telephone treatment aligns with wider health policy (Petrova & Dale, 2006) around patient choice and flexibility, patient choice was not always at the centre of decisions to offer telephone at service level, demonstrating mistranslation of principles from policy level to practice.

#### Cognitive participation: engagement with telephone-delivered psychological interventions

Successfully implementing telephone interventions into existing practice in IAPT is reliant in part on the engagement or commitment of all stakeholders – both decision makers and front line staff – to new ways of working. Whilst benefits of telephone working were acknowledged by all participants, a significant number of barriers to engagement remain.

The extent to which teams and services commit to telephone working depends on willingness to change, and by how well organisations support staff to make changes. Decision makers acknowledged the difficulty in instigating change, identifying staff confidence and skill-set as important factors. The impact of this was evidenced by one participant who spoke about a tendency to ‘drift’ to old ways of working (counselling) in services where staff had previously worked in alternative roles. This created the need to remodel telephone working in some services to correct this:

*‘There’s been within our service a culture, sort of, a bit reluctant to take on telephone work among staff but we’ve had to, sort of, re-focus step two quite a bit anyway in terms of the way that they’re working because I think there’s been historically quite a bit of drift from guided self-help.’ (Participant 13, local informant, emergent use)*

Willingness of patients to engage was also an essential factor identified by decision makers, which could be affected by staff attitudes to telephone treatment. Individual experiences of decision makers appeared to influence perspectives of telephone treatment, in particular where they had previously worked as a clinician delivering treatment. In addition, there was a perception amongst decision makers of the need to ‘sell’ telephone interventions to patients, which was a challenge in itself when it was felt there was first a need to ‘sell’ telephone to PWPs:

“*I mean I really think it's about capturing hearts and minds, not just of patients but of the therapists….If they don’t believe in it themselves they can't talk about it appropriately to the patient because they can't sell it, and if you can't do that then you're already on to a loser.” (Participant 6, local informant, established use)*

Given the impact of personal attitudes towards the use of telephone treatments, such views must be considered in terms of local implementation. Despite understanding the drivers for telephone treatments at policy level, individual attitudes could play a disproportionate role in local variability in the absence of national guidance and standards.

#### Collective action: implementing telephone-delivered psychological interventions into practice

Collective action is the work that needs to be undertaken by individuals and organisations to successfully implement new procedures and practices. Decision makers were able to identify the work that is needed and the resources and staff skills required to make telephone treatment work in practice.

As discussed above in terms of perception and attitudes, the need to action ‘selling’ of telephone interventions to PWPs was one issue emphasised by decision makers. Incentivising PWPs to make telephone treatment more appealing was the strategy identified for this task, accomplished by providing opportunities such as flexible working, working from home and greater autonomy over managing caseloads. The success of incentivising even just a small number of staff with this flexibility was also identified as having a subsequent benefit to raising acceptability amongst the wider workforce:

*‘One lever is giving people the opportunity to work at home which some people really like, I mean, it depends on peoples personal circumstances obviously but for some people that works well. So, you know, that’s an incentive, a useful lever for some……As staff have begun to take on telephone work and have had something positive to say about it, that’s been useful for other staff.’ (Participant 13, local informant, emergent use)*

The need to incentivise PWPs was highlighted to be in part related to a lack of skills and confidence, and suggested as being resultant of insufficient inclusion of telephone skills in many PWP training courses. Decision makers identified a significant shortfall in training for working on the telephone, where policy has failed to influence the curriculum. During the focus group with local decision makers, many of whom had completed the PWP course, numerous participants reported experiencing limited training on telephone work, such as a single slide or presentation at University. To address this, many organisations represented by the decision makers had developed or commissioned their own in-house training on telephone working to address skills gaps in the workforce. One participant spoke about the service ‘boot camp’ in which PWPs are intensively trained in telephone working when they commence their role, and subsequent on-going monitoring of practice.

The gap in skill set observed after PWP training was also felt in part by some to be related to the highly prescriptive, protocol-driven approach of the national PWP curriculum, which could be at the expense of teaching the skills needed for practice due to the lack of inclusion of telephone working:

*“Certainly the courses that our PWPs go to…it does seem to be heavy on the protocol, heavy on quite a rigid protocol adherence to pass the course, as opposed to the good old-fashioned, idiosyncratic formulation and working within the models that fits within that formulation. It seems to be...I hate to say it, but almost like a cookbook approach to training…So essentially it means that when we have therapists come and join us we invariably are spending a fair bit of time working with them to use telephone working, with mixed results really.” (Participant 3, local informant, emergent use)*

In addition to training and incentives, the importance of investment in resources to support telephone working was evident, in particular where services have adopted telephone working as a ‘quick fix’. Issues around IT provision and specific tools to aid telephone treatment were noted as important practical considerations. In addition there was acknowledgement of the essential necessity of basic equipment and suitable working environment for PWPs, but that sometimes such resources are not provided:

*“So, the availability for therapists of appropriate equipment that they would need to do the job. So, things like headsets, a quiet space to do the phone calls, things like that, these are often the things that are the last down the list and sort of seem to be a wish list item rather than a necessity, and yet I think that to do the job properly you need the things that are going to help you do that.” (Participant 6, local informant, established use)*

Potential obstacles with how face to face procedures are undertaken in telephone sessions, such as completion of outcome measures and engaging in written tasks for treatment, were also discussed. Participants talked about the issue of wasting valuable treatment time when completing measures, and the potential for the development of coercive relationships, where patients feel under pressure to report feeling better than they do due to the on-the-spot nature of completing measures by telephone during the session. Additional practical barriers were identified by decision makers, particularly difficulties resulting from a lack of visual communication, such as undertaking formulations during assessments:

*‘One of the problems that we still face with telephone assessments is doing things like drawing out formulations. So, if you're drawing something with a client that's virtually impossible over the phone.’ (Participant 5, local informant, limited use)*

#### Reflexive monitoring: appraisal and future sustainability of telephone-delivered psychological interventions

All participants agreed on the validity and long term sustainability of telephone treatments; that they will remain a treatment option in order to provide accessible interventions to patients. Some were keen to ensure that telephone interventions remained part of a wider offer, noting that the modality will not suit all and choice of treatment should remain the central tenet of IAPT provision. Use of IAPT minimum datasets was suggested by decision makers as one way to monitor efficiency and recovery rates, but difficulties with this and the need for improvements to the dataset itself were noted. Issues regarding the accuracy of reporting mode of delivery for the IAPT dataset were highlighted, along with the current lack of facility for services to extract data specifically for telephone treated patients:

*‘I don't think we have a robust way yet of monitoring the vehicle that therapy is delivered by, be that telephone treatment, be that a digitally enabled therapy, because the dataset doesn’t accurately gather that information and we don’t report on it accurately enough, which is why we are looking at revisions to the IAPT dataset so we can much more accurately capture how that's delivered and sort of demonstrate the outcomes for different methods of delivery.’ (Participant 6, local informant, established use)*

Interestingly, the suggestion of IAPT dataset use by a local informant contrasted against a national decision maker’s suggestion of using patient feedback data, which appears reflective of the misaligned drivers for telephone at the local and national level described above. Using data available to measure services against key performance indicators, compared to the emphasis of using a measure more greatly focussed on patient experience, demonstrates the divergence in priority between higher level and local decision makers.

A change in recording of telephone contacts in clinical record systems was additionally highlighted as a potential determinant of how PWPs might alter their views of the value of telephone treatment. Some Systems were known to not have the facility to record telephone treatment sessions in the same way, or be given the same weight as face-to-face sessions, potentially contributing to the perception of PWPs that face-to-face delivery is superior:

*‘It’s still very difficult for some contacts to be considered as contacts [on the] clinical recording system and given the same weight as…even if those telephone contacts are therapeutic in their delivery’. (Participant 1, national informant)*

Additional approaches identified for improving acceptability of telephone treatment included measures to address PWP concerns and anxieties. Decision makers discussed approaches such as providing the opportunity for PWPs to observe telephone working in practice, and the introduction of service ‘champions’ who could advocate for the benefits of telephone treatments:

*“I think you have certain people…when you’ve got people around who champion it and talk about the relative merits that it might offer for a service, but outside of that, you know, that tends to be the exception rather than the rule.” (Participant 1, national informant)*

The influence of individuals advocating for telephone, demonstrated to be effective by some services already, further indicates the disproportionate effect a single opinion within a service can have in the absence of best practice guidance for IAPT services.

All decision makers commented on the need for a combination of monitoring, PWP training, advocacy for the use of telephone, and the need for clearer guidelines for implementation in order to achieve a more standardised approach to telephone across IAPT services.

## Discussion

This study aimed to explore the perspectives of local and national decision makers on the implementation of telephone-delivered treatment in IAPT services. Despite acknowledgment of the clinical, practical and service benefits of telephone treatments, patient engagement is often not maintained [21], raising the question as to why this is so. Exploring the implementation of telephone delivered treatments in IAPT services from multiple angles including insights from high level local and national decision makers, PWPs at the clinical interface and patients receiving treatment, is crucial in drawing a global picture to identify key issues to address Data from this study will be combined with findings from associated independent studies of patient and PWP perspectives of telephone delivered treatment (reported elsewhere) to inform the development of a quality improvement intervention for the delivery of telephone treatments, and enhancement of patient engagement and outcomes, as part of a wider researcher programme [28].

Data suggest that the importance of telephone treatment is recognised at local and national level as having clinical benefit in terms of providing patient choice and flexibility, but that this can lead to tensions when the drive to provide patient-centred psychological treatment is pitted against organisational pressures related to performance, targets and funding. Misalignment between national and local level objectives, views and opinions on the way telephone treatments can be implemented and monitored was evident across all four NPT constructs. While the same barriers and facilitators to implementation were identified by both local and national decision makers, understanding of the practical implications posed by telephone delivery was evidently diverged. Importantly, this appears to have a downstream impact at the clinical level. Failure of policy to influence the curriculum has also led to insufficient training for PWPs in the delivery of telephone treatments, which has created an issue of confidence across the workforce. This, combined with sceptical views of the organisational motivations for the implementation of telephone treatments has generated the perception of the need to incentivise the workforce in order for telephone treatment to then be ‘sold’ to patients. Acknowledgement of the necessity to invest in quality training and resources and guidance on implementation was highlighted as essential, both for buy-in from staff and practical implementation in services.

### Strengths and limitations of the study

This study gains strength from its robust in-depth qualitative approach, which combined thematic analysis with NPT to enhance and frame the data. The data sample spanned a variety of key individuals across IAPT to provide a rich picture of the national IAPT landscape. The study was limited by the number of high-level key individuals available to interview, and our relatively limited snowball method of sampling. Interviews were based on personal opinions by decision makers in relation to attitude of others in the implementation chain which might not represent the true landscape. However, as individual opinion appears an important factor in relation to the study, we are confident the data is sufficient to inform our interpretations.

### Comparisons with other studies

Although there is a large literature indicating the difficulties in implementing and sustaining new practices and technologies in healthcare [35, 36] evidence exists to demonstrate the potential for clinical and cost effectiveness of telephone treatment [15]. A recent systematic review and meta-analysis of the impact of telephone delivered case management for depression in collaborative care demonstrated no loss of effectiveness compared to face to face case management [37] which supports our findings for the drivers for the provision of telephone treatment in IAPT. In addition, a study of patient acceptability of telephone CBT for widespread pain highlighted similar considerations around acceptance and the benefits of telephone treatment for managing felt stigma [38]. Organisational tensions, misaligned drivers to meet targets and issues around inadequate training for PWPs highlighted by decision makers in this study are reflective of previous accounts of IAPT [39], and discussion around cost-saving and efficiency are in agreement with other literature [40].

### Implications of the findings

The IAPT model is an idiosyncratic context where a relationship based treatment is delivered in a managed environment using fairly prescriptive models. We have used NPT to explore the current setting and to understand the tensions that exist. Data from this study will be combined with findings from associated studies of patient and PWP perspectives of telephone delivered treatment (reported elsewhere) to inform the development of a quality improvement intervention for the delivery of telephone treatments, and enhancement of patient engagement and outcomes, as part of a wider researcher programme [28].

Data in the current study has identified misalignment between national policy makers and local decision makers. While there is common understanding of the benefits offering telephone treatment can provide patients, understanding why and how telephone is used across services diverges between national and local leads. The tendency for emphasis to be weighted towards the perception of telephone as an efficiency measure at service level can have an impact on attitudes of PWPs, which can have negative consequences. Targeting those issues that impact on PWP perceptions within any proposed intervention will be important.

Important factors clustered around cognitive participation centre around decision makers’ perspectives on the confidence of PWPs to deliver treatments over the telephone, and subsequent willingness to change working practices. The need to incentivise PWPs to adopt telephone working was key in terms of collective action and was central in discourse at both local and national level. Acknowledgement that once telephone is ‘sold’ to PWPs, they in turn must then ‘sell’ it to patients, and the challenges associated with that in terms of PWP coherence, will potentially be an essential target of our intervention. Work within our wider research programme may shed interesting light on whether PWP views around incentives are indeed as important and influential as perceived by decision makers.

Our results suggest that our intended intervention will have to engage with both beliefs and attitudes and skills enhancement. Data gathered within the reflexive monitoring component of the NPT framework also indicated that steps to incorporate feedback and monitoring information for improving the acceptability of telephone work for PWPs will also be important. IAPT has a clear focus on the use of data to support service delivery – but this is focussed on service outcomes, defined externally. Such outcomes may meet the expectations of external observers, but it may be important that they also allow staff delivering treatments to understand the impacts of increased telephone delivery on outcomes they value, such as patient experience and the quality of the relationships they are able to maintain.

The intervention will have to work at multiple levels, ensuring that it impacts on local service managers (as well as PWPs), and that the impacts on PWPs are explicitly designed to encourage them to overcome concerns that might be present in patients.

A review using NPT which assessed published systematic reviews of professional behaviour change interventions suggested that interventions more likely to be successful are focussed on the constructs of collective action and reflexive monitoring: ‘participants in successful behaviour change interventions may have responded positively to a clear sense of how what they were asked to do made sense (its coherence), and how their actual responses to this (their collective action) measured up to the expectations of external observers (reflexive monitoring)’ [41]. Our intervention will have a significant focus on actual behaviour, informed by relevant psychological theory (the theoretical domains framework, [24, 42]) and also by a detailed analysis of actual therapeutic conversations (through application of conversation analysis), with outputs relevant to both practice and education.

## Conclusions

Guidance on best practice for implementation and operationalization for organisational leads, along with the necessity to invest in and provide adequate support for PWPs in terms of training and provision of resources, is important for the successful implementation of telephone treatment in IAPT services.

## Supplementary information

**Additional file 1.** Interview Schedule.

## Data Availability

The dataset generated and analysed during the current study are not publicly available due to potential for breach of anonymity, but are available from the corresponding author on reasonable request.

## Abbreviations

CBT: Cognitive Behavioural Therapy
DBT: Dialectical Behaviour Therapy
EMDR: Eye Movement Desensitisation
HIT: High Intensity Therapy
IAPT: Increasing Access to Psychological Therapies
NHS: National Health Service
NICE: National Institute of Health and Care Excellence
NPT: Normalisation Process Theory
PWP: Psychological Wellbeing Practitioner
RCT: Randomised Controlled Trial
