# Correction to: A case of misalignment: the perspectives of local and national decision-makers on the implementation of psychological treatment by telephone in the Improving Access to Psychological Therapies Service

**DOI:** 10.1186/s12913-020-4895-2

**Published:** 2020-01-14

**Authors:** Kelly Rushton, Claire Fraser, Judith Gellatly, Helen Brooks, Peter Bower, Christopher J. Armitage, Cintia Faija, Charlotte Welsh, Penny Bee

**Affiliations:** 1 School of Health Sciences, Division of Nursing, Midwifery and Social Work, University of Manchester, Manchester Academic Health Science Centre, Manchester, UK; 2 Department of Health Services Research, Institute of Population Health Sciences, University of Liverpool, Liverpool, UK; 3 NIHR School for Primary Care Research, Centre for Primary Care and Centre for Health Informatics, Manchester Academic Health Science Centre, University of Manchester, Manchester, UK; 4 School of Health Sciences, Division of Psychology and Mental Health, University of Manchester, Manchester University NHS Foundation Trust, Manchester Academic Health Science Centre, Manchester, UK; 5 School of Health Sciences, Division of Psychology and Mental Health, University of Manchester, Manchester Academic Health Science Centre, Manchester, UK

**Correction to: BMC Health Serv Res**

**https://doi.org/10.1186/s12913-019-4824-4**

In the original publication of this article [1], the article title should be revised as blow:

A case of misalignment: the perspectives of local and national decision-makers on the implementation of psychological treatment by telephone in the Improving Access to Psychological Therapies Service

This error is caused due to a typesetting mistake.

The original article has been corrected.
