# Supplementary material for: A case of misalignment: the perspectives of local and national decision-makers on the implementation of psychological treatment by telephone in the Improving Access to Psychological Therapy is the name of the service and should be capitalised
Source: BMC Health Serv Res. 2019 Dec 26;19:997. doi: 10.1186/s12913-019-4824-4 (PMC6933680; doi:10.1186/s12913-019-4824-4)
Supplement: Supplementary file 1 — Additional file 1. Interview Schedule. [file 12913_2019_4824_MOESM1_ESM.docx]

**Additional File 1**

**Interview Schedule**

**(A): Local Service Managers/Clinical Leads**

1. Can you please tell me a little bit about your role in the delivery of IAPT services?
2. To what extent is providing telephone-delivered psychological interventions part of your IAPT service?

- How is that decision made? By whom?
- What is driving that decision? Policy – local and/or national level?
- How well do you think policy takes account of telephone interventions? It is under/over-represented why?

1. If telephone services are offered – What is the current target population?

- Criteria based or service wide offer? Who gets it?

1. If telephone services are offered – Can I ask you to reflect on the implementation of telephone-delivered interventions locally?

- Have there been any challenges?
- What has gone well?
- How does it fit with existing local service provision for psychological interventions?
- How do you think offering telephone-delivered psychological interventions has impacted on ways of working in the IAPT service?
- Are resources available to support therapists?
- Are there any incentives to promote use?
- How is quality assessed? How does this impact on service delivery?

1. Do you have any sense of the patient perspective on telephone-delivered psychological interventions?

- Perceived benefits and risks?
- Are there any particular types of patients for whom telephone-delivered psychological intervention is more helpful/difficult?

1. What are your views on the long term sustainability of telephone-delivered psychological interventions?

- Perceived benefits and risks?
- Can you identify any barriers to sustainability?
- Any suggestions about future development of the local service?
- Where do telephone therapies best sit? Which step? Which services? Why?
- What would need to be in place to encourage diffusion to other steps/services?

1. What advice would you have for other areas wishing to implement telephone-delivered psychological interventions locally?
2. Is there anything else that we have not covered that you would like to add?
3. Can you think of anyone else who it might be useful for me to speak to?

**(B): National/Policy Leads**

1. Can you please tell me a little bit about your role and how this relates to delivery of IAPT services?
2. What role do you think telephone-delivered psychological interventions should play in the IAPT programme?
3. How well do you think policy takes account of telephone interventions? It is under/over-represented why?
4. Is there any scope for expansion and or development of telephone interventions in IAPT?

- In what way?
- What systems need to be in place to support/encourage this?

1. Where do telephone therapies best sit? Which step? Which services? Why?

- What would need to be in place to encourage diffusion to other steps/services?

1. What are your views on the long term sustainability of telephone-delivered psychological interventions?

- Perceived benefits and risks?

1. What are the major barriers to development of telephone interventions in IAPT?

- Evidence base?
- Workforce?
- Policy?
- Management models?
- Patient demand?

1. Is there anything else that we have not covered that you would like to add?
2. Can you think of anyone else who it might be useful for me to speak to?
